# A multidimensional ODE-based model of Alzheimer’s disease progression

**DOI:** 10.1038/s41598-023-29383-5

**Published:** 2023-02-23

**Authors:** Matías Nicolás Bossa, Hichem Sahli

**Affiliations:** 1 Department of Electronics and Informatics (ETRO), Vrije Universiteit Brussel (VUB), 1050 Brussels, Belgium; 2 Interuniversity Microelectronics Centre (IMEC), 3001 Leuven, Belgium

**Keywords:** Alzheimer's disease, Computational models, Alzheimer's disease, Statistics

## Abstract

Data-driven Alzheimer’s disease (AD) progression models are useful for clinical prediction, disease mechanism understanding, and clinical trial design. Most dynamic models were inspired by the amyloid cascade hypothesis and described AD progression as a linear chain of pathological events. However, the heterogeneity observed in healthy and sporadic AD populations challenged the amyloid hypothesis, and there is a need for more flexible dynamical models that accompany this conceptual shift. We present a statistical model of the temporal evolution of biomarkers and cognitive tests that allows diverse biomarker paths throughout the disease. The model consists of two elements: a multivariate dynamic model of the joint evolution of biomarkers and cognitive tests; and a clinical prediction model. The dynamic model uses a system of ordinary differential equations to jointly model the rate of change of an individual’s biomarkers and cognitive tests. The clinical prediction model is an ordinal logistic model of the diagnostic label. Prognosis and time-to-onset predictions are obtained by computing the clinical label probabilities throughout the forecasted biomarker trajectories. The proposed dynamical model is interpretable, free of one-dimensional progression hypotheses or disease staging paradigms, and can account for the heterogeneous dynamics observed in sporadic AD. We developed the model using longitudinal data from the Alzheimer’s Disease Neuroimaging Initiative. We illustrate the patterns of biomarker rates of change and the model performance to predict the time to conversion from MCI to dementia.

## Introduction

There is an increasing interest in data-driven disease progression models (DPM) of Alzheimer’s disease (AD) because of their potential application in disease mechanism understanding, development of clinical prediction models of diagnosis and prognosis, and clinical trial design. The rate of change of biomarkers, cognitive tests, and clinical measurements, and their relationship with other variables, are estimated to understand the dynamics and internal mechanisms of the disease or to test hypotheses^1,2^. DPMs can also be used as the backbone of clinical prediction models, particularly for progressive diseases such as AD, where the clinical condition worsens over time. In these cases, clinical prediction models aim to predict the speed of progression and time to symptom onset. Moreover, DPMs are helpful for clinical trial design and simulation^3^. For example, they can help to identify the subjects with faster progression, reducing the costs associated with sample size or trial duration.

AD progression models were often inspired by the amyloid cascade hypothesis, crystallised into Jack’s hypothetical model of biomarker dynamics, which states that the main AD biomarkers become abnormal in a temporally ordered manner^4,5^. Even though there is a large consensus that A$\beta$ plays a critical role in AD pathophysiology^6^, growing evidence shows that AD progression comes from a multifactorial interaction of processes^7^ and that all combinations of biomarker abnormalities are frequently present in the cognitively normal population^8,9^. Furthermore, dementia can be caused by multiple pathologies, and AD often co-occurs with them, especially after age 65^10,11^. The A$\beta \text {/tau/neurodegeneration}$ (AT(N)) framework, a biomarker-driven classification system that makes no assumptions about the order in which the biomarkers become abnormal, was proposed to define and stage AD across its entire spectrum^12^. More recently, a new conceptual model of AD^13^ posited a non-deterministic disease path. According to this model, A$\beta$ and tau levels interact between them and with genetic and environmental factors to increase or reduce the risk of disease progression. These interactions would be responsible for the heterogeneity observed in biomarker trajectories and the discrepancies between observations and the amyloid cascade hypotheses.

Precise quantitative tools that estimate the biomarker dynamics are needed to shed light on the AD process and to build better clinical tools for diagnosis, prognosis and therapy efficacy assessment. These tools must accurately predict cognitive decline and determine which combination of biomarkers produces faster disease development. The AT(N) framework and the probabilistic model of AD^13^ could help to build more interpretable and informative models.

### Disease progression models of Alzheimer’s disease

Quantitative models of AD can be divided into (i) traditional (regression models), which explore associations between variables; (ii) clinical prediction models, aimed for diagnosis and prognosis prediction; and (iii) DPMs. DPMs could be further divided into dynamical and event-based models, which assume discrete jumps from normality to abnormality to discover the sequence of events. The distinguishing feature of dynamical DPMs is that they allow for quantifying and predicting the temporal evolution of the relevant dynamical variables.

The first AD progression models describing long-term trajectories from short-term biomarker observations were based on Jack’s model^4^, i.e., that all subjects follow the same disease progression pattern but with different onset times and speeds. Jedynak defined a disease progression score aimed at quantifying disease progression and therapeutics’ effectiveness^14^. Subjects were temporally ordered according to this score, and a parametric sigmoid-shaped curve was used to fit the progression of biomarkers. In^15^, the authors proposed a semi-parametric model to determine the population mean of biomarker trajectories and the temporal order of subjects. A similar but more flexible model, proposed by^16^, used Gaussian Process (GP) to model also the individual departures from the mean. In general, all these models may suffer from identifiability issues when trained with short-term observations because of the need to simultaneously estimate the disease onset times and the biomarker trajectories. Identifiability issues were usually mitigated using mixed-effect modelling to restrict the variance of the subject-level parameters.

The first dynamical model that relaxes the *unique trajectory* hypothesis, allowing an arbitrary combination of variables as initial conditions, used a Riemannian framework to transport the mean trajectory to fit the subject’s observations^17–19^. Contrary to the previous works, and similarly to our proposal, it is the basal level of the variables, not the onset time, which was modelled as the subject-level parameter.

Finally, differential equation models parameterise biomarker velocities instead of biomarker trajectories. They are, therefore, *implicit* models. Some works^20,21^ tackle the problem of estimating long-term biomarker trajectories from short-term observations of a single biomarker. A recent work^22^ used a system of ordinary differential equations (ODE) to simulate the effect of amyloid treatments on the disease course.

In this work, we propose a probabilistic DPM with the following characteristics: (i) no assumptions of biomarker time-order or typical disease trajectory across individuals; (ii) smooth trajectories of biomarker and cognition features implicitly modelled through systems of differential equations; (iii) modelling of covariate effect on trajectories; and (iv) the ability to work with arbitrary measurement schedules, missing data, and unsynchronised observation times of different biomarkers. Our model has certain similarities with the work of^17^ because all combinations of trajectory basal levels are allowed. But it is also an extension of the differential equation models because velocity is the key modelling feature. In particular, our model is conceptually very similar to^22^, the most notable differences being the mathematical implementation and the focus on the causal interpretation. Specifically, our model is more straightforward because we model the progression speed directly on the observed biomarkers without the need to define latent variables; it is more general because we do not prescribe a specific functional form for the velocities; our paper focuses on the prediction of progression, while in^22^, the authors interpret their model from a causal perspective.

### An ODE-based model of disease progression

We propose a generative (semi)parametric Bayesian AD evolution model that admits several progression paths. It has the flexibility to handle heterogeneous acquisition times, follow-up lengths, and missing data. The main idea is to model the rate of change of dynamical variables (biomarkers and cognitive tests) as a function of their current values and other relevant variables, i.e., using a system of ordinary differential equations (ODE). In this work, we opted for a linear ODE (given by Equation (1) in the “Method” section). However, other functions can be used to describe the velocity. For example, Gaussian Processes were used in^23^, and a second-order polynomial was used in^22^.

**Figure 1:**
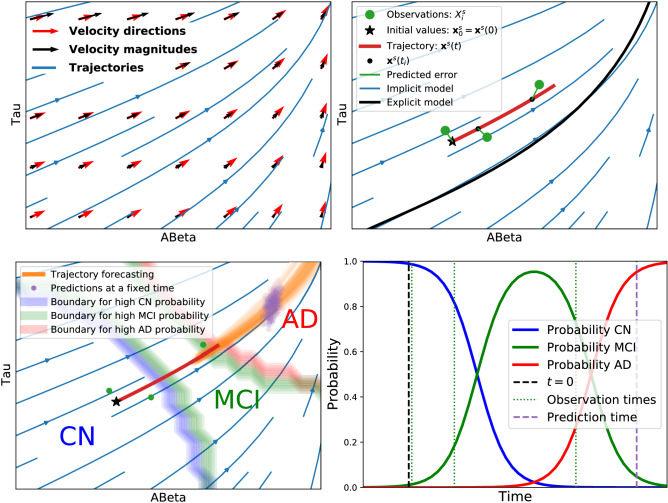
Schematic representation of the proposed framework for AD progression modelling. Top left: A velocity field describes the biomarker evolution at any point in biomarker space. Velocity direction quantifies how each biomarker is changing relative to the others, and velocity magnitude quantifies the mean yearly rate of change. Long-term trajectories are obtained starting at a given point in the biomarker space and evolving with speed equal to the velocity field evaluated at each point. Top right: The modelled trajectories (blue curves) can pass through any point in the biomarker space, contrary to explicit models that describe one-dimensional trajectories (black curve). Individual predictions are obtained by fitting the most probable subject trajectory (red curve), i.e. the closest trajectory to the observations (green dots) at the correct time points. Only the initial value (black star) needs to be estimated. Then the trajectory is computed according to the velocity field. The initial value estimation has no serious identifiability issues because it should be close to the observations at the corresponding reference time, the left-most green dot in this case, regardless of the velocity field estimation. On the contrary, explicit models estimate the time-to-onset or an equivalent parameter that locates the individual’s trajectory on the black curve. These two parameters (mean trajectory and individuals’ time-to-onset) are highly correlated, giving rise to identifiability issues. Bottom left: A clinical prediction model assigns a clinical label probability for any combination of biomarkers and covariates. For a fixed set of covariates, these probabilities can be visualised as a heat map. The figure in this panel illustrates the boundaries where the most likely label changes, from Cognitively Normal in the bottom left extreme (normal levels in both biomarkers) to Dementia in the top right extreme (abnormal levels in both biomarkers). These boundaries depend on the subject covariate values. Therefore, they are located at different places for each person. The subject trajectory can be forecasted following the velocity field (orange curves). The purple dots represent the predicted biomarker levels at a fixed time after the last observation, along with their uncertainty. We can estimate future clinical outcomes using these forecasted values as input to the clinical prediction model. Bottom right: Probability for each clinical label along the predicted subject trajectory as a function of time. Vertical lines denote the initial time (black), observation times (green) and the selected time for prediction given by the purple dots in the bottom left panel (purple). Note that in the biomarker space representations (the top panels and the bottom left panel), there is no explicit indication of the speed at which the biomarker space is being travelled and of predicted diagnosis label change in time. The length of trajectory segments at different regions of the biomarker space is not indicative of the relative times needed to traverse them.

Our modelling strategy is linked to a significant paradigm shift that abandons the idea of a natural temporal order of biomarker abnormalities^24^. Suppose some events happen earlier in the course of the disease. In that case, it should be reflected in the joint distribution of all variables, showing that only a small fraction of the space of dynamical variables is occupied. However, it is not an *a priori* assumption. Figure 1 illustrates the main elements of the proposed approach.

Compared to other DPMs^16,19^, the disease evolution is described here from a different perspective. The fixed point in our framework is the value of the biomarkers and not the time at diagnosis. In this work, we raise the question “what is the most likely biomarkers evolution and the probability of having a dementia diagnosis at a given time in the future given the current state?” and not “what is the most likely combination of biomarkers at a given time before and a given time after the dementia diagnosis?”. In our model, we consider the possible existence of a surface in the biomarker space corresponding to different combinations of biomarkers levels at the time of dementia diagnosis. Therefore, the biomarker levels at a given time before crossing this boundary depend on the point at which the patient crosses it (see bottom left panel of Fig. 1). Not learning a canonical order of biomarkers allows us to focus on two important tasks: understanding the biomarkers dynamics, and clinical prediction. i.e., to model how the current combination of biomarkers levels and covariates affect the rate of change of the biomarkers on one side and to predict the risk of developing dementia in the future on the other.

## Results

### Disease progression

**Figure 2:**
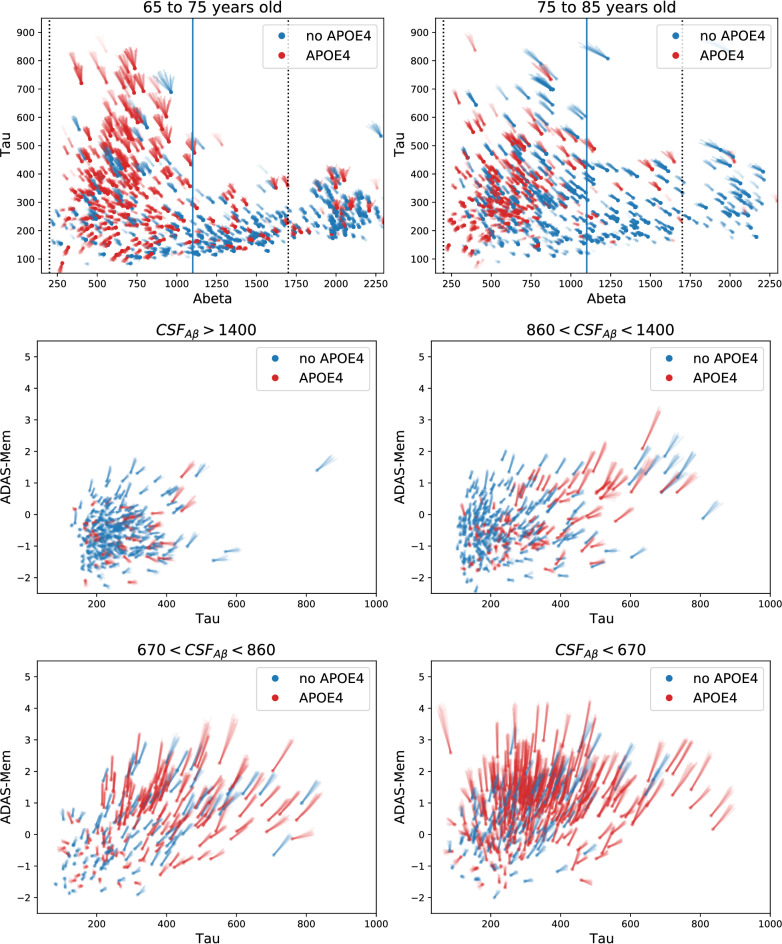
Velocity fields. Dots represent mean estimated initial values (i.e., basal level) and lines represent velocity (two years of evolution) and their uncertainty (lines are drawn from the posterior distribution). Top: CSF A$\beta$ and Tau biomarkers. The left (right) panel shows younger (older) subjects. The solid vertical line at CSF A$\beta = 1100$ denotes the usual threshold of abnormality. Dotted vertical black lines show detection limits. A$\beta$ values beyond these limits were estimated based on the censored likelihood, random effect components of the model, and consistency with the velocity field. The uncertainty of the initial A$\beta$ value for these subjects (not represented in the figure) is much larger than for the rest. Middle and bottom: Memory trait of ADAS-Cog and CSF Tau for different levels of CSF A$\beta$. Larger ADAS-Mem denotes larger ADAS-Cog scores and, therefore, worse cognitive performance. Cut-offs for CSF A$\beta$ (shown in the panel titles) corresponds to the $25\%$, $59\%$, and $75\%$ percentiles.

The disease evolution is encoded in the velocity field. Figure 2 shows velocity field maps on different axes only on regions populated with observations, i.e., each point corresponds to an individual from the training set. These plots inform qualitatively about the biomarker distribution and the rate of change in the population. For completeness, complete trajectories for each subject are shown in the Supplementary Materials (Fig. S2). However, trajectories are less informative than velocity maps because the length of trajectories depends on the follow-up duration, which is different for each subject.

The top panels of Fig. 2 show the velocity in the CSF A$\beta \text {-Tau}$ plane. Note that lower CSF A$\beta$ is associated with higher brain A$\beta$ load. The younger subjects (top-left) present a more usual AD pattern, which may be interpreted as a slow increase of brain A$\beta$ followed by an increase of Tau. Progression accelerates when both amyloid and Tau are high. APOE4 subjects already have large brain amyloid (red dots on the left of top panels), which may be explained by an early start of the amyloid accumulation process that begins before the minimum age represented in this subset of the ADNI population (mainly above 60 years old). High amyloid levels are not necessarily followed by a Tau accumulation, particularly for APOE4 subjects, as reflected by a large proportion of subjects with elevated amyloid, low Tau, and low rate of change (see short segment lines at the bottom-left corner of both top panels). The pattern seems more heterogeneous for older patients (top-right panel), perhaps due to concomitant pathologies producing tau accumulation in this group, expressing a less pure AD physiopathology. In addition, there is a strong survivorship bias among the older subjects because the most severe cases already died or were not included in the study following the ADNI protocol.

Cognition dynamics are represented in the middle and bottom panels of Fig. 2. Velocities are shown in the cognition-Tau plane for different levels of A$\beta$. One of the most salient features is that the cognition decline accelerates for low CSF A$\beta$ (bottom-right panel) regardless of Tau status, especially for APOE4$\epsilon 4$ subjects. However, for intermediate levels of amyloid load, there appears to be a correlation between Tau and the rate of cognitive decline. In summary, a fast cognitive decline requires either that both amyloid and Tau are abnormal or that CSF A$\beta$ is very low.

**Figure 3:**
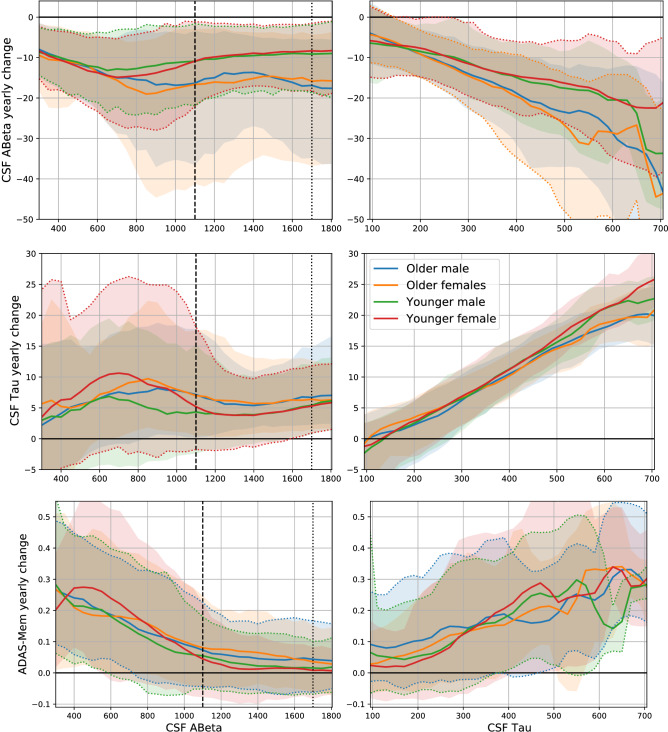
Biomarker (Top and middle) and cognition (Bottom) rates of change for different biomarker basal levels (CSF A$\beta$ on the left and CSF Tau on the right). Shaded regions show the expected velocity distribution (90% HDI posterior probability) for the ADNI population conditioned on CSF A$\beta$ or CSF Tau.

Figure 3 shows the rate of change of the CSF biomarkers and cognition as a function of the CSF A$\beta$ (left) and CSF Tau (right) basal levels, including the age and gender effects. Remarkably, CSF Tau is a precise predictor of both Tau and A$\beta$ progression (top and middle right panels). In contrast, the relation of A$\beta$ with biomarker dynamics is less evident (top and middle left panels). Low CSF A$\beta$ is correlated with faster progression, but a large proportion of subjects with abnormal CSF do not show biomarker change. Only A$\beta$ presents a plateau effect (see Fig. 3 left side of top left panel), a pattern frequently reported^1^. Regarding cognition (bottom panels), the opposite pattern is observed, both CSF biomarkers are correlated with cognition decline, but Tau presents a higher variability. Figure 3 illustrates how the model could be used for hypothesis testing or rate of change quantification. After the research question is formulated, the appropriate parameter could be estimated from these predictions.

Many works have investigated the biomarker dynamics in AD using standard statistical tools. Specifically, linear regressions are first computed for each subject to obtain the individual rates of change, followed by another regression to estimate how the biomarker rates of change vary with the basal biomarker levels. Contrary to these methods, the proposed model can provide the posterior probability distributions of the velocities marginalised in the population and conditioned on the biomarker levels and covariates of interest. Our model can be used to address specific hypotheses, such as the association between biomarkers levels and their rates of change, in a single coherent analysis that accounts for all uncertainty sources. For example, the rate of increase of PET tau and PET amyloid has been studied in subjects from different USA and Sweden cohorts^25^, including, but not exclusively, ADNI participants. They “found that the tau accumulation rate is greater in females and younger amyloid-$\beta$-positive individuals, while amyloid-$\beta$ accumulation is greater in APOE E4 carriers and older individuals”^25^. A similar pattern can be observed in the middle-left panel of Fig. 3 (there is a higher probability of larger rates of tau accumulation among A$\beta$ positive subjects compared to A$\beta$ negative subjects, but this difference is more significant for younger females), and top panels of Fig. 3 (only younger males and females present the largest rate of A$\beta$ accumulation). Note that the shaded regions represent the posterior probability of velocity values in the ADNI dataset. It is not the confidence interval of the mean, which would require further model assumptions.

A more recent work^26^ on CSF and PET tau of subjects from the USA, not including ADNI participants, reported that “higher CSF p-tau181, lower CSF A$\beta$42, and higher amyloid PET levels were associated with faster rates of tau PET change”^26^. Even though the tau-related biomarker studied in our work was CSF total Tau, and not PET nor phosphorylated Tau, the middle right panel Fig. 3 shows a strikingly linear association between tau rates of change and basal levels. Similar patterns were observed in^27^, where the implications of using fluid biomarkers for screening in clinical trials testing tau-targeting therapies were highlighted.

### Clinical prediction

#### Discrimination

**Figure 4:**
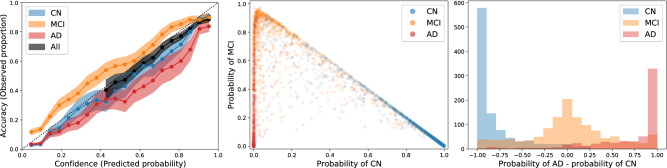
Clinical predictions in the leave-one-site-out cross-validation experiment on the whole ADNI dataset. For each left-out subject, a prediction was estimated at each diagnosis assessment time after the first two years of follow-up. Left: Calibration estimation. The dots represent the mean (shaded areas the 95% CI) of the confidence- (black) and classwise-reliability diagrams (coloured). Centre: Simplex plot illustrating the mean predicted probability assigned to each observation (e.g., the probability corresponding to the points lying on the bottom-left corner is $100\%$ AD, $0\%$ MCI and $0\%$ CN). The dot colour indicates the ground truth label. Right: Histograms of the predicted AD probability minus the CN probability for each ground truth clinical label.

The discrimination power was high (AUC $\sim 0.92$ for AD vs non-AD), which is not surprising given that CN, MCI, and AD populations are already very different and a small proportion changes their diagnosis during the follow-up. The histograms of the predicted probabilities vs the ground truth label can be observed in the centre and right panels of Fig. 4. CN are almost perfectly separated from AD.

#### Calibration

In addition to discrimination, well-calibrated probabilities are another desired feature of clinical prediction models. Calibration refers to the agreement between predictions and observed outcomes. It is critical in clinical decision-making^28^, yet it is often overlooked when reporting the results of clinical prediction models.

A standard tool for measuring calibration is given by the calibration plots (or reliability diagrams in Machine Learning literature). They consist in plotting the accuracy or proportion of positive outcomes as a function of the predicted probability^29^. A diagonal line indicates that the predictions are perfectly calibrated. For multi-class prediction, the equivalent concepts of *confidence-reliability diagram* and *classwise-reliability diagrams* were recently defined^30^. The former is the overall accuracy (proportion of observations for which $\text {argmax}( \hat{ \textbf{p}} )$ is the correct class) for each probability $c \in [0, 1]$ (i.e., $\max (\hat{ \textbf{p}} )=c$), and the latter is the accuracy for each class as a function of the probability assigned to this class.

The left panel of Fig. 4 shows the confidence- and classwise-reliability diagrams with different colours (top-left). The shaded regions represent the 95% CI computed as the posterior of a Beta-Binomial model at 0.2 width bins (the larger the bins, the smaller the uncertainty but also the resolution). Confidence-reliability is reported only for probabilities larger than 0.4 because $1/3$ is the minimum attainable value for the maximum probability for three classes.

### Prediction of MCI conversion to AD

Discrimination of MCI converters from non-converters and time-to-conversion are more challenging but valuable tasks. In this section, we show the model performance only for MCI subjects who did not convert to AD before the first two years of follow-up.

#### Discrimination accuracy

**Figure 5:**
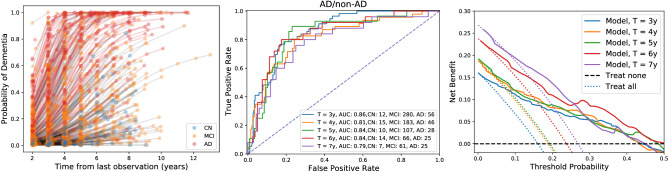
Prediction of conversion from MCI to AD as a function of time from baseline in the cross-validation experiment. Left: Probability assigned to the AD label as a function of time. Dots show the diagnosis assessments (the colour denotes the diagnosis), and lines connect assessments of the same subject. Red lines are used for subjects with an AD diagnosis at any point during the follow-up (converters), and grey lines are used for the rest (non-converters). The blue dots correspond to subjects who reversed their diagnosis to CN after being MCI during the first two years. Centre: Accuracy (ROC curves) of the conversion prediction (AD/non-AD classification) at specific times after baseline. There is at most one diagnosis assessment per subject each year, and the number of subjects is reduced for longer follow-ups (see the legend). Right: Decision curves. The AD prevalence equals the intersection of the “Treat all” strategy with the “Treat none” strategy. The model is useful only for probability thresholds for which the Net Benefit is greater than for the “Treat all” and “Treat none” strategies. The probability threshold depends on the risks and benefits of the therapy under consideration.

To visualise how diagnosis evolves in time for each subject, the left panel of Fig. 5 shows the probability of AD as a function of time, along with the diagnosis assessment of each MCI subject. Probability increased with time for most subjects but at a faster rate for converters (red dots denoting a dementia diagnosis). Note that all these subjects had MCI diagnoses exclusively during the first two years of follow-up.

The area under the receiver operating characteristic curve (AUROC) was 86, 84 and 79 for conversion prediction at 3, 5 and 7 years after baseline, respectively (see the centre panel of Fig. 5).

#### Decision curve analysis

Decision curve analysis is used to evaluate the utility of clinical predictions through net benefit plots^31^. The risk-benefit ratio of a given medical action could be reduced to a threshold probability $p_{th}$ defined as follows. If $p_{th}$ were the true probability of the subject having the disease, then the benefits of taking action (e.g., recommend a treatment) when the patient is AD would compensate for the harms of taking action when the patient is not AD. The threshold probability is fixed for a given setting (i.e., a disease and a chosen medical action). It considers the expected medical improvement and side effects in patients, the harm to healthy people, the economic costs, and any other quantifiable factors. The decision curve tells us how better (or worse) it is to treat all patients, treat none or treat only the ones with a risk larger than the threshold ($P(AD)>p_{th}$) according to a prediction model^29^. The right panel of Fig. 5 shows the net benefit when the model is used to predict the probability of conversion to AD a certain number of years after the first visit. The intersection of the *treat all* curves with the *y* or *x*-axis reflects the increasing prevalence of the number of converters with time. The model showed that prognostic predictions are useful for a wide range of threshold probabilities, up to $50\%$. The leftmost part of the graph corresponds to situations where the harm of not intervening is high, whereas the rightmost side corresponds to dangerous interventions with little benefit.

#### Time to AD conversion

Another way of evaluating AD conversion prediction performance, and digging deeper into model behaviour, is to look at the time to AD conversion. The error is now measured in the time discrepancy between the predicted and observed conversion time. To define the predicted conversion time, we should define an AD probability threshold at which we consider the subject has converted. We restricted the analysis to the population subset having MCI diagnoses during the first two years of follow-up and at least two CSF measurements in the same period.

**Figure 6:**
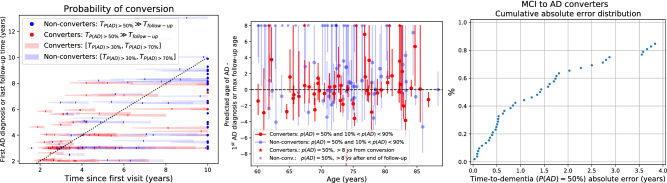
Predicted time to AD conversion for subjects enrolled with an MCI diagnosis who do not convert to AD during the first 2 years of follow-up. Left: Time from the first visit to the first AD diagnosis (for converters, in red) or maximum follow-up time (for non-converters, in blue) is shown in the$y$-axis. Shaded regions show the time when the predicted probability of AD is larger than $30\%$ (left end) up to $70\%$ (right end), with a mark denoting the time corresponding with a $50\%$ AD probability. The dots on the right of the figure represent subjects whose predicted time of conversion is larger than 10 years. Centre: In red it is shown the time to conversion error (predicted minus actual time of conversion) vs age for converters. In blue is shown the difference between the predicted age of conversion minus the maximum follow-up time for non-converters. Right: Cumulative absolute error distribution for converters.

The left panel of Fig. 6 shows the predicted time to AD conversion for converters (red) and non-converters (blue). On the $y$-axis is the time of the first AD diagnosis for converters and the maximum follow-up time for non-converters. Ideally, the red marks (converters) should lie on the diagonal (black dotted line), which means that the model predicted precisely the time to AD conversion. On the other hand, all the blue marks should lie on the right of the diagonal, which means that the model did not predict a conversion before the last evidence of no conversion. The centre panel shows the error in predicted time to conversion for converters (in red) and the difference between predicted time to conversion and max follow-up for non-converters (in blue) as a function of age. Ideally, the red dots should lie on the horizontal dashed line (no error) and the blue dots above that line. The right panel of Fig. 6 shows the cumulative distribution of the time-to-conversion absolute error for the 52 patients who converted after the first two years of follow-up and had two CSF measurements in this period.

As a rough comparison, time-to-onset was studied for dominantly-inherited AD subjects in^23^. Contrary to our multidimensional model, the authors applied a differential equation model to each biomarker independently. Then, a weighted average of the estimated time-to-onset was computed and compared with the actual date of onset for a subset of six participants who converted during the follow-up. A root mean squared error of 1.34 years was reported for these six subjects. In our experiments, the error in time-to-conversion was higher, less than 2 years for $66\%$ of the subjects, which is equivalent to 1 std. if the error distribution were Gaussian (see the right panel of Fig. 6). However, these numbers must be compared carefully because of many reasons. First, episodic and dominantly-inherited AD may have different dynamics. Additionally, many biomarkers (in addition to CSF) were used in^23^, which should help to obtain more precise predictions. The onset time was defined as having non-zero global CDR in their work, which would correspond to CN to MCI conversion, while we studied conversion from MCI to dementia. Finally, the sample sizes are very different, with six subjects in^23^ and 52 in our work. Nevertheless, we remark that the errors in both cases are of the same order of magnitude.

### Long-term trajectories

**Figure 7:**
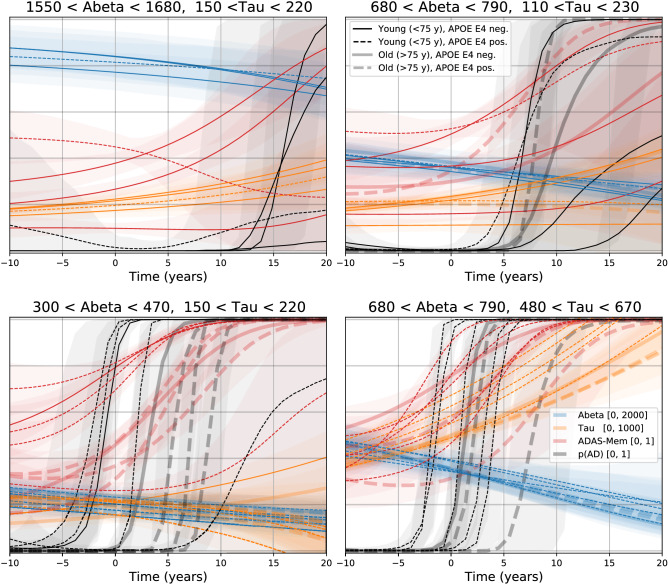
Simulated long-term evolution for different initial conditions. Subjects for whom the basal CSF biomarker levels were within limits described on top of each panel and with a smaller uncertainty were selected. Then, trajectories were propagated 10 years backwards and 20 years forwards, taking into account the subject’s parameters and the model uncertainty. The y-axis range for each biomarker is indicated in the legend at the bottom right panel.

We simulated long-term trajectories for a few sets of representative subgroups to compare our predictions with those of dynamical^15^ and event-based models^23,32^. We selected 4 points in the CSF A$\beta$-Tau plane: normal, medium-high, and high A$\beta$ with low Tau (see the top-left, top-right and bottom-left panels of Fig. 7, respectively), and medium-high levels of A$\beta$ with very high Tau (see the bottom-right panel of Fig. 7). All the subjects within each subgroup have similar values of CSF biomarkers, but ADAS-Cog, age and APOE may differ, giving rise to different trajectories.

A few remarks could be drawn from these patterns: CSF biomarker dynamics are much slower than cognition (CSF biomarkers do not reach a plateau in any of these panels); clinical diagnosis and cognition onset time is highly variable between subjects; CSF Tau increases rapidly only when CSF A$\beta$ is abnormal, but also there must be already high levels of CSF Tau (see the bottom-right panel compared with the bottom-left panel); very abnormal levels of CSF A$\beta$ predict cognitive decline and dementia even in the absence of CSF Tau (see the bottom-left panel).

## Discussion

We have presented a DPM that helps to overcome three important limitations of previous progression models of AD^33^. Remarkably, the proposed framework dispenses with the assumption of a common disease trajectory, allowing to build quantitative models in line with the modern ATX(N) conceptual framework^34^. Biomarker independence is another limiting assumption frequently made. Conversely, the relationship between one biomarker level and another biomarker dynamics is the core of the proposed framework. Finally, we consider the effect of covariates in the dynamics, i.e., the fixed effects, to be an integral part of our model.

We have illustrated a concrete implementation of a DPM and shown its potential as a tool for AD dynamics understanding and clinical prediction. We decided to build the most elementary but still meaningful model of AD progression. However, the model could be improved and expanded in many directions, and many questions still need to be answered. The most obvious and straightforward steps are the inclusion of more biomarkers, such as MRI features, CSF phosphorylated Tau (p-Tau), Amyloid and Tau PET, FDG PET, and A$\beta$ in blood, among others. Note that the model does not extend trivially with the number of biomarkers because the dimension of the matrices describing the velocities increases with them. Adding more biomarkers may require carefully designing the matrix (6) structure or replacing the velocity parameterisation with a non-parametric model. Both options could be computationally challenging. Additionally, a more exhaustive validation in other datasets different from ADNI and following standardised evaluation protocols^35^ is needed for a fair comparison with other approaches.

The predicted biomarker and cognition dynamics were only described qualitatively because we were not trying to answer any particular research question in that regard. The model could be used in different ways for quantitative analysis and hypothesis testing. For example, a contrast could be defined, such as the difference between the mean velocity of subjects with two sets of characteristics, and the posterior of this difference can be explored. Also, subjects included in the model training should be carefully selected to discard the presence of confounders or other sources of bias in the parameter estimations.

We have not illustrated applications of the model to clinical trial design, but we identify at least two elements that could benefit from DPMs. Given an expected treatment effect on an outcome measure, the model could be used to simulate the evolution of a specific population and estimate the expected change and variability of the outcome measure on the untreated group. Next, we can optimise the clinical trial costs by selecting the sample size or follow-up duration so that the change in the untreated patients is large enough to detect the treatment effect. Alternatively, we can change the population characteristics to identify the subgroup where the target outcome changes the fastest. In^22^, the authors use their model to simulate the effect of amyloid treatments on the disease course. We do not recommend using our model for this type of causal prediction because we did not do any causal analysis. We believe, for example, that the fact that subjects with different levels of a given biomarker consistently show a different rate of cognitive decline doesn’t mean that externally modifying the process associated with this biomarker would stop that decline. By simulating the progression of a given population, we are not predicting the effect of any treatment or external action, we are forecasting the natural disease progression.

Similar to our model, the normative model described in^17–19^ also allows modelling trajectories that can pass through an arbitrary biomarker combination at a given point in time. However, some major conceptual differences can be highlighted. The normative model builds a reference trajectory of all the biomarkers. Even though the order in which the biomarkers become abnormal can be changed arbitrarily to fit a given subject data, some trajectories will be closer and others further from the reference pattern. In our model, no trajectory can be considered a reference or template. In^17–19^, the speed of progression could vary even for two subjects following the same trajectory. This flexibility allows an even greater variety of patterns to be accommodated but requires repeated measurements to adjust the individual speed. In our model, the speed of progression depends only on the biomarker levels and the covariate of interest, allowing predicting progression even with cross-sectional data. Furthermore, as we explicitly model the biomarker rates of change as a function of the basal biomarker levels and the covariates, we can describe how each variable is associated with the rate of change of each biomarker, which are magnitudes of interest in themselves.

Despite these limitations, the proposed framework provides qualitative advantages over previous approaches, offering new possibilities, even if the reported performance were not optimal. The rate of change of biomarkers and cognitive tests and its relationship with basal biomarker levels, age and genetic information was previously extensively studied to unveil the internal mechanisms that drive the disease^25,36–39^. A common approach is to fit a linear or cubic model to data points in the velocity-intercept plane^1,2^. This is a two-step process because velocities are not directly observed, and the uncertainty in the velocity estimations is not propagated to the subsequent analyses. These questions could be easily articulated within the proposed model, which provides a testbed that estimates velocities and allows statistical inference in a single coherent statistical setting. The advantage is that the velocity field produces a principled regularisation consistent with the observed trajectories. That is, the observed rate of change should be the same as the velocity of the predicted trajectory, a consistency constraint not always considered.

## Methods

### Statistical setting

The proposed statistical model comprises three key components: an evolution model of an internal state (dynamical) variable, a likelihood function linking the observed biomarkers and cognitive tests with the state variable, and an instantaneous clinical prediction model.

Let $\textbf{x}(t)$ be a set of *dynamical variables* tracking the course of the disease, linked to some biomarkers, cognitive tests, or other measurements, and $\textbf{y}$ be a set of relevant covariates that may affect the disease evolution or manifestation, such as sex, age, therapeutic interventions, or genetics. The model relies on the assumption that the rate of change at a given point in time depends only on the current values of the dynamical variables and covariates. There are no assumptions about the number and observation times of each biomarker, neuropsychological and clinical assessment. Then, the evolution of the disease can be described by a system of ODEs

1 $$\begin{aligned} \frac{d \textbf{x}(t)}{dt} = \textbf{v} (\textbf{x}(t), \textbf{y} ). \end{aligned}$$

The trajectory $\textbf{x}^s(t)$ for a given subject $s$ is given by the solution to the ODE (1) with initial condition $\textbf{x}^s(0) = \textbf{x}_0^s$

2 $$\begin{aligned} \textbf{x}^s(t) = \textbf{x}_0^s + \int _0^t \textbf{v}(\textbf{x}^s(\tau ), \textbf{y}^s) d\tau . \end{aligned}$$

The velocity field $\textbf{v}( \cdot , \textbf{y})$ describes the mean disease evolution process for each sub-population (given by the levels of the covariate $\textbf{y}$), and $\textbf{x}_0^s$ is the only subject-level parameter, which determines the complete time evolution of the dynamical variables for the subject $s$. Integral (2) can be analytically solved for a few special cases. Otherwise, it should be approximated numerically.

Let $k$ identify a biomarker or a cognitive test and $X^s_{i, k}$ be its observation at time $t^s_{i, k}$. The number and observation times for each subject $s$ and measurement type $k$ could be different and are indexed with $i$. A subset of the dynamical variable vector, $\textbf{x}^s_k(t)$, is linked to this measurement $X^s_{i, k}$ via some likelihood function,

3 $$\begin{aligned} X^s_{i, k} \sim \mathfrak {L}_k( \textbf{x}^s_k(t^s_{i,k}), \Theta _k ) \end{aligned}$$

In other words, $\textbf{x}^s_k(t)$ is the predicted smooth trajectory, and $X^s_{i, k}$ the corresponding noisy observations, which could belong to spaces with a different number of dimensions. $\Theta _k$ are the corresponding likelihood parameters, such as noise variance. $\mathfrak {L}_k$ could be a Gaussian distribution in the case of scalar biomarkers, a more complex model for imaging features, or an Item Response Theory (IRT) model for neuropsychological assessments.

Another likelihood function predicts the probability of a diagnostic label in terms of the dynamical variables and covariates. It could be anything from simple logistic regression to a more complex model. Let $D^s_j$ be a clinical assessment at time $t^s_{j, \text {Clin}}$, then the clinical prediction model is given by

4 $$\begin{aligned} D^s_j \sim \mathfrak {L}_D( \textbf{x}^s(t^s_{j, \text {Clin}}), \textbf{y}^s, \Theta _D ). \end{aligned}$$

The description above is the core of the proposed method. The model may also include a hierarchical structure described in Supplementary Material Description S1.2.

### Statistical model Alzheimer’s disease

Following the framework described above, we present a minimal concrete model of Alzheimer’s disease progression. In this work, we included only two biomarkers, CSF A$\beta _{1-42}$ and CSF total Tau, and one cognitive test, ADAS-Cog. The likelihoods for the CSF biomarkers were Gaussian distributions. A right-censored model was used for A$\beta _{1-42}$ because the assay has a prescribed upper detection limit of 1700 pg/mL. To model cognition, we used a multidimensional Item Response Theory (IRT) based scoring methodology^40^ that extracts latent traits of three cognitive domains: language, memory, and praxis (see Supplementary Material, Description S1.1). Therefore, the dynamical variable $\textbf{x}$ is a 5-dimensional vector $\textbf{x} = [x_{\tau }, x_{A\beta }, x^{\text {Cog}}_{\text {Lang.}}, x^{\text {Cog}}_{\text {Mem.}}, x^{\text {Cog}}_{\text {Praxis}}]$. Only two covariates were included, $y_1 = \text {age}$, and $y_2 = \text {APOE4}$, represented as a binary value indicating whether there is at least one copy of the $\epsilon 4$ allele of the apolipoprotein-E (APOE) gene.

The outcomes for the clinical prediction model were the following three diagnostic labels: Cognitive Normal (CN), Mild Cognitive Impaired (MCI) and Dementia (AD). As MCI is regarded as an intermediate phase between normal ageing and dementia, we used an ordered logit model for the diagnosis likelihood (4). The predictors were the vector of dynamical variables $\textbf{x}(t)$ evaluated at the diagnosis time together with the vector of covariates $\textbf{y}$.

#### Dynamical model

A common assumption of AD DPMs is that all biomarkers and cognitive tests change slowly and monotonically at the time scale we are considering (a few decades). This is justified by the biological and cognitive processes involved and the frequency of biomarker sampling in long-term studies, which could be annual in the best case. For these and for computational reasons, we parameterise $\textbf{v}(\cdot )$ linearly. Note that, in more than one dimension, a linear velocity does not necessarily imply exponential growth. In addition, we used a sigmoid shape function to link the dynamical variable $\textbf{x}(t)$ with all the observations, allowing a plateau effect at the observation level. An additional advantage of linear velocity fields is that the ODE can be solved explicitly using the matrix exponential, reducing the computational burden compared with numerical integration methods. Specifically,

5 $$\begin{aligned} \textbf{v}( \textbf{x}, \textbf{y} ) = \left( V + \sum _{c \in \{1,2\}} y_c W_c \right) \textbf{x} + \textbf{v}_0, \end{aligned}$$

where $V$, $W_c$ and $\textbf{v}_0$ are model parameters.

Even though we are not making causal claims about variables and model parameters, we expect that the biomarker velocities are not affected by the cognitive status. We can therefore reduce the number of model parameters without compromising the prediction performance by setting to zero some components of the velocity matrices $V$ and $W_c$,

6 $$\begin{aligned} V = \begin{pmatrix} V_{\text {CSF}} &{} \textbf{0} \\ V_{\text {CSF,Cog}} &{} V_{\text {Cog}} \end{pmatrix}. \end{aligned}$$

#### Priors and hyperpriors

We set a hierarchical prior on the initial values $\textbf{x}_0^s$, with Gaussian hyperpriors and weakly informative hyperpriors for the population means and variances. See Supplementary Material Description S1.2 for details.

### Experimental setting

#### Participants

The model was fitted to the Alzheimer’s Disease Neuroimaging Initiative (ADNI) database (adni.loni.usc.edu). The ADNI was launched in 2003 as a public-private partnership, led by Principal Investigator Michael W. Weiner, MD. The primary goal of ADNI has been to test whether serial magnetic resonance imaging (MRI), positron emission tomography (PET), other biological markers, and clinical and neuropsychological assessment can be combined to measure the progression of mild cognitive impairment (MCI) and early Alzheimer’s disease (AD). All ADNI participants provided written informed consent, and study protocols were approved by each local site’s institutional review board. Ethics committees/institutional review boards that approved the study are: Albany Medical Center Committee on Research Involving Human Subjects Institutional Review Board, Boston University Medical Campus and Boston Medical Center Institutional Review Board, Butler Hospital Institutional Review Board, Cleveland Clinic Institutional Review Board, Columbia University Medical Center Institutional Review Board, Duke University Health System Institutional Review Board, Emory Institutional Review Board, Georgetown University Institutional Review Board, Health Sciences Institutional Review Board, Houston Methodist Institutional Review Board, Howard University Office of Regulatory Research Compliance, Icahn School of Medicine at Mount Sinai Program for the Protection of Human Subjects, Indiana University Institutional Review Board, Institutional Review Board of Baylor College of Medicine, Jewish General Hospital Research Ethics Board, Johns Hopkins Medicine Institutional Review Board, Lifespan-Rhode Island Hospital Institutional Review Board, Mayo Clinic Institutional Review Board, Mount Sinai Medical Center Institutional Review Board, Nathan Kline Institute for Psychiatric Research & Rockland Psychiatric Center Institutional Review Board, New York University Langone Medical Center School of Medicine Institutional Review Board, Northwestern University Institutional Review Board, Oregon Health and Science University Institutional Review Board, Partners Human Research Committee Research Ethics, Board Sunnybrook Health Sciences Centre, Roper St. Francis Healthcare Institutional Review Board, Rush University Medical Center Institutional Review Board, St. Joseph’s Phoenix Institutional Review Board, Stanford Institutional Review Board, The Ohio State University Institutional Review Board, University Hospitals Cleveland Medical Center Institutional Review Board, University of Alabama Office of the IRB, University of British Columbia Research Ethics Board, University of California Davis Institutional Review Board Administration, University of California Los Angeles Office of the Human Research Protection Program, University of California San Diego Human Research Protections Program, University of California San Francisco Human Research Protection Program, University of Iowa Institutional Review Board, University of Kansas Medical Center Human Subjects Committee, University of Kentucky Medical Institutional Review Board, University of Michigan Medical School Institutional Review Board, University of Pennsylvania Institutional Review Board, University of Pittsburgh Institutional Review Board, University of Rochester Research Subjects Review Board, University of South Florida Institutional Review Board, University of Southern, California Institutional Review Board, UT Southwestern Institution Review Board, VA Long Beach Healthcare System Institutional Review Board, Vanderbilt University Medical Center Institutional Review Board, Wake Forest School of Medicine Institutional Review Board, Washington University School of Medicine Institutional Review Board, Western Institutional Review Board, Western University Health Sciences Research Ethics Board, and Yale University Institutional Review Board. All methods were carried out in accordance with relevant guidelines and regulations. Further information about ADNI, including full study protocols, complete inclusion and exclusion criteria, and data collection and availability can be found at adni.loni.usc.edu.

**Table 1:** Counts of subjects and observations by baseline diagnosis and gender.

Diagnosis	CN		SMC		EMCI		LMCI		AD	
Gender	F	M	F	M	F	M	F	M	F	M
Subjects	145	139	56	39	125	155	142	221	96	137
Age^a^	74(6)	75(6)	72(5)	72 (5)	70(8)	72 (7)	72 (7)	75 (7)	73 (8)	76 (8)
CSF^b^	326	325	86	54	201	252	293	489	147	197
ADAS-Cog^b^	960	937	244	167	757	937	876	1386	310	425

^a^Age in years, mean (std), ^b^number of observations.

All subjects from the ADNI dataset with at least one CSF observation were included, resulting in 1255 participants. The baseline characteristics of the population included in the analysis are summarised in Table 1. The mean(maximum) number of CSF and ADAS-Cog observations per subject were 1.9(8) and 5.6(16), and the mean(maximum) follow-up durations were 1.5(10) and 4(15) years, respectively.

#### Model fitting and validation

Inference of model parameters was made using the Stan software^41^. The Stan code is available in Supplementary Material (Fig. S3). Internal-external cross-validation has been used to evaluate the model performance using a leave-one-site-out experiment. This validation design is more efficient than split sample approaches and a better approximation to external validity than leave-one-out designs^42^. At each iteration, a site was selected for testing and the rest for training. All the information from the first 2 years of each patient belonging to the left-out centre was used to forecast the rest of the follow-up, and the procedure was repeated for all centres.

## Supplementary Information

Supplementary Information.

## Data Availability

Data used in preparation of this article were obtained from the ADNI database (http://adni.loni.usc.edu). The model code is available in Supplementary Material (Fig. S3).
