# Supplementary material for: A multidimensional ODE-based model of Alzheimer’s disease progression
Source: Sci Rep. 2023 Feb 23;13:3162. doi: 10.1038/s41598-023-29383-5 (PMC9950424; doi:10.1038/s41598-023-29383-5)
Supplement: Supplementary file 1 — Supplementary Information. [file 41598_2023_29383_MOESM1_ESM.pdf]

Supporting Information: A multidimensional ODE-based model of Alzheimer's disease progression

Matías Nicolás Bossa and Hichem Sahli

S1 Supplementary Descriptions

S1.1 IRT model

The ADAS-Cog is discrete and bounded, which points to the binomial distribution as the simplest model. However, this model is not the most appropriate because all the rich information from individual sub-items is lost. Therefore, we used an Item Response Theory (IRT) based scoring methodology ([Verma et al., 2015]) that extracts latent traits of three cognitive domains. 12 of the 13 items that ADAS-Cog are divided into three traits: language, memory and praxis. For each of the 12 items, we used ordered logistic models, *a.k.a.* proportional odds ([McCullagh, 1980]) or Samejima's graded response model (GRM) in this context ([Samejima, 1968]), where the three latent scores are shared among a predefined set of items. The model proposed in [Verma et al., 2015] used GRMs for graded responses (*e.g.*, mild/moderate/severe) or counts (*e.g.*, number of recalled words) and three-parameter logistic (3PL) models for items composed of dichotomous scores (*e.g.* month, season or place from the Orientation category). In contrast, we used GRM for all the items for simplicity.

Let $A_p \in \{1, \ldots, K_p\}$ be the score of the *p-th* ADAS-Cog item, whose possible values are integers from 1 to the number of levels (K_p) of that item, then the likelihood is defined by:

$$
\begin{aligned}
P(A_p = 1) &= 1 - \text{logit}^{-1}\left(-(x_{\text{Cog},I_p}\alpha_p - d_{p,1}\right) \\
P(A_p > k) &= \text{logit}^{-1}\left(-(x_{\text{Cog},I_p}\alpha_p - d_{p,k}\right) \\
&\vdots \\
P(A_p = K_p) &= \text{logit}^{-1}\left(-(x_{\text{Cog},I_p}\alpha_p - d_{p,K_p-1}\right)
\end{aligned}
$$

where α_p is called the item discrimination or scale parameter, and $\{d_{p,k}\}_{k=1}^{K_p-1}$ are the item difficulty or location parameters (see [Verma et al., 2015]). There are, therefore 3 continuous dynamical variables linked to ADAS-Cog, $x_{\text{Cog,lang.}}$, $x_{\text{Cog,mem.}}$ and $x_{\text{Cog,pra.}}$, each associated with a different cognitive domains. The index variable I_p indicates to which of the three cognitive domains belongs each item p.

The likelihood of an individual score k is known as the item characteristic function

$$P(A_p = k) = P(A_p > k - 1) - P(A_p > k).$$

Figure S1 shows the posterior distribution of the item characteristic functions fitted to the ADNI dataset.

S1.2 Prior distributions and hierarchical structure

In a Bayesian setting, we should choose priors for the parameter Θ_k, Θ_D, $\mathbf{v}(\cdot)$ and $\mathbf{x}_0^s$ (see equations (2), (3) and (4) in the main text), that can be a fixed probability distribution or a parameterized probability distribution. The latter case gives rise to a so-called hierarchical or multilevel structure ([Gelman et al., 2013]) and has different uses related to regularization, shrinkage and smoothing (see [Hodges, 2013], chapter 13).

The following two sets of parameters in the proposed model may benefit from a hierarchical structure. Firstly, the velocity field $\mathbf{v}(\cdot)$ can be modelled parametrically or non-parametrically (*e.g.*, with Gaussian Process priors). The non-parametric models often require a multilevel structure, for example, priors on the covariance parameters. Note that this choice will determine which methods can be used to integrate or approximate the ODE. Secondly, the subject-level parameters (*e.g.*, $\mathbf{x}_0^s$) can be treated as a random effect, which is standard practice for all longitudinal studies, among many others.

One benefit of including a hierarchical structure to subject-level parameters is that it can handle naturally missing observations, which are assumed completely at random in this work. Missing data can be treated as unknown parameters if we want their posterior distribution to impute values on other models or simply for prediction purposes. If there were known missing data mechanisms in a dataset (*e.g.*, missing not at random), additional likelihood terms could be easily included. These terms should model the distribution of observed and missing values in terms of the appropriate covariates ([Gelman et al., 2013]).

We set the following hierarchical prior on the initial values $\mathbf{x}_0^s$

$$x_{0,\tau}^s \sim \mathcal{N}(\mu_\tau, \rho_\tau) \quad (1)$$
$$x_{0,A\beta}^s \sim \mathcal{N}(\mu_{A\beta}, \rho_{A\beta}) \quad (2)$$
$$\mathbf{x}_{0,Cog}^s \sim \mathcal{N}(\mathbf{0}, \Omega), \quad (3)$$

with normal ($\mathcal{N}(0,1)$) hyperpriors on CSF mean parameters μ_τ and $\mu_{A\beta}$, and half normal prior on CSF variance parameters ρ_τ and $\rho_{A\beta}$. These hyperpriors are very weakly informative since CSF biomarkers were already transformed to take values between 0 and 1.

The cognitive part of $\mathbf{x}_0^s$ requires special attention to avoid identifiability issues associated with the IRT models. Adding an arbitrary constant vector to all $\mathbf{x}_{0,Cog}^s$ is equivalent to subtracting the same vector components to the appropriate location parameters $d_{p,k}$. The same happens with variance and scale parameters α_p. One common approach to achieve identification is to set the latent variable mean to 0 and the variance to 1, as shown in eq. 3, where Ω is a correlation matrix. We let the model have the freedom to learn the correlation terms of the covariance matrix. The Lewandowski-Kurowicka-Joe (LKJ) distribution is usually recommended for correlation matrices ([Gelman et al., 2013])

$$\Omega \sim \mathrm{LkjCorr}(1), \quad (4)$$

where the shape parameter equal to 1 implies that all the correlation terms have a uniform distribution.

S2 Supplementary Figures

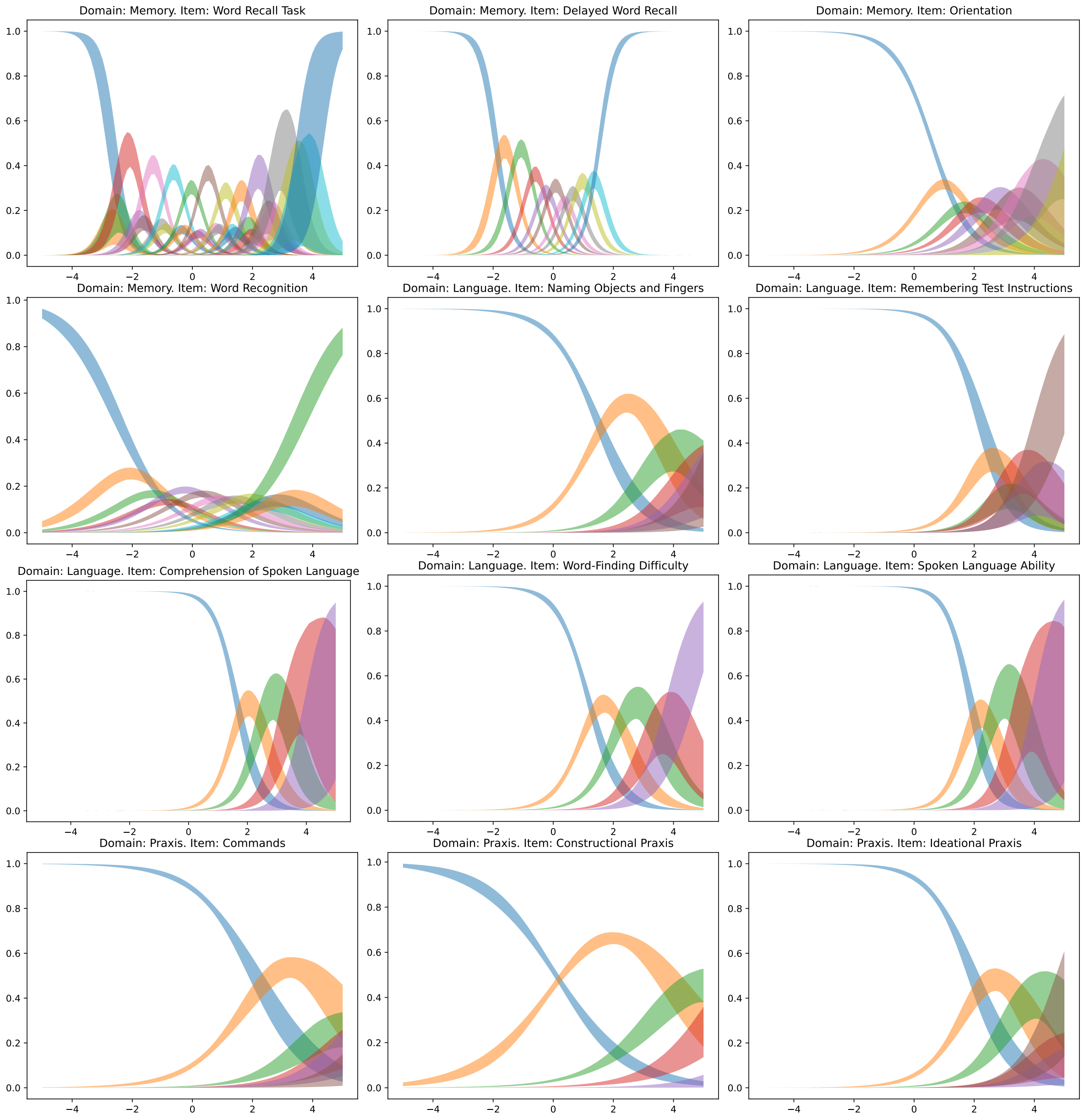

Figure S1: Posterior distribution of the item characteristic functions.

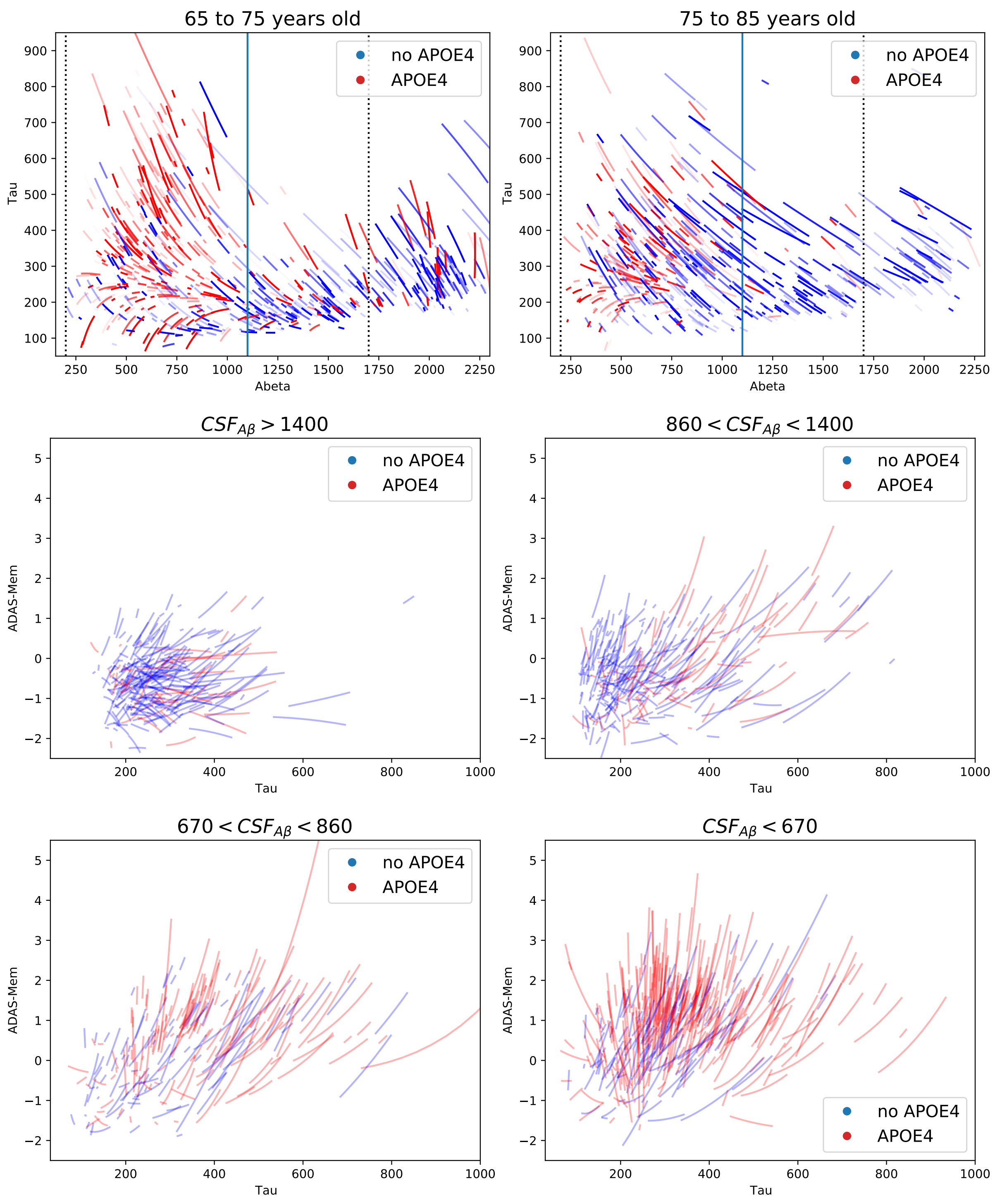

Figure S2: Estimated individual mean trajectories. Note that the curve lengths are not related to the biomarker rate of change but to the subject's follow-up duration.

S3 Model specification

```
data {
  int N;                              // Number of subjects
  int n;                              // Number of observations
  real t[n];                          // Observation times
  int m;                              // Numer of covariates
  matrix[n, m] X;                     // Covariates
  real AGE[N];                        // Subjects' age
  int<lower=0, upper=1> APOE4[N]; // One APOE4 allele minimum
  int<lower=1, upper=N> ID[n];    // Subj. ID for each observation
  int kTau;            // Number of elements of Tau vector (1 or 2)

  // Number of valid observations for each type of data
  int nAB;
  int nTau;
  int nADAS;
  int nDX;

  // Indexes to locate time (t) or ID for each measurement
  int<lower=1, upper=n> idxAB[nAB];
  int<lower=1, upper=n> idxTau[nTau];
  int<lower=1, upper=n> idxDX[nDX];
  int<lower=1, upper=n> idxADAS[nADAS];

  // Observations
  matrix[nTau,kTau] Tau;
  vector[nAB] AB;
  int<lower=1, upper=3>   DX[nDX];
  // ABETA censored info
  real<lower=0, upper=1> ABmin;
  int<lower=0, upper=n> NABCens;
  int<lower=1, upper=n> idxABCens[NABCens];
  // ADAS
  int<lower=2> ADASCats[12];        // Number of categories per item
  int<lower=1, upper=ADASCats[ 1]>  Q1[nADAS]; // categorical data
  int<lower=1, upper=ADASCats[ 2]>  Q2[nADAS];
  int<lower=1, upper=ADASCats[ 3]>  Q3[nADAS];
  int<lower=1, upper=ADASCats[ 4]>  Q4[nADAS];
  int<lower=1, upper=ADASCats[ 5]>  Q5[nADAS];
  int<lower=1, upper=ADASCats[ 6]>  Q6[nADAS];
  int<lower=1, upper=ADASCats[ 7]>  Q7[nADAS];
  int<lower=1, upper=ADASCats[ 8]>  Q8[nADAS];
  int<lower=1, upper=ADASCats[ 9]>  Q9[nADAS];
  int<lower=1, upper=ADASCats[10]>  Q10[nADAS];
  int<lower=1, upper=ADASCats[11]>  Q11[nADAS];
  int<lower=1, upper=ADASCats[12]>  Q12[nADAS];
  int<lower=1>  D;                // Number of dimensions/factors
  int<lower=1, upper=D> it_d[12]; // Which factor corresponds to each item
}
transformed data {
  // Auxiliary variable with zeros
  int kCSF = kTau + 1;
  int K = kCSF + D; // Total number of features
  matrix[kCSF, D] zCSF = rep_matrix(0, kCSF, D);
}
parameters {
  vector[N] c0CSF[kCSF];        // Random effects (CSF)
```

```
  real<lower=0> sRECSF[kCSF];   // Variance of CSF RE
  real muRECSF[kCSF];           // Mean CSF

  // Velocity field parameters (main effect and interactions)
  matrix<lower=-0.2, upper=0.2>[kCSF,kCSF] vCSF;
  matrix<lower=-0.5, upper=0.5>[D, K] vADAS;
  row_vector[K] v0;
  matrix<lower=-0.1, upper=0.1>[kCSF, kCSF] wCSFAge;
  matrix<lower=-0.1, upper=0.1>[kCSF, kCSF] wCSFAPOE;
  matrix<lower=-0.2, upper=0.2>[D, K] wADASAge;
  matrix<lower=-0.2, upper=0.2>[D, K] wADASAPOE;

  // Observation noise variances
  real<lower=0> sTau[kTau];
  real<lower=0> sAB;

  // Prediction model parameters
  vector<lower=-20>[K + m] bDX;     // Predictor coefficients
  ordered[2] cDX;                   // Cut-off points

  // ADAS-Cog IRT model parameters
  vector<lower=0, upper=5>[12] alpha; // Slopes for each item
  ordered[ADASCats[ 1]-1] th1;    // Thresholds for each item
  ordered[ADASCats[ 2]-1] th2;
  ordered[ADASCats[ 3]-1] th3;
  ordered[ADASCats[ 4]-1] th4;
  ordered[ADASCats[ 5]-1] th5;
  ordered[ADASCats[ 6]-1] th6;
  ordered[ADASCats[ 7]-1] th7;
  ordered[ADASCats[ 8]-1] th8;
  ordered[ADASCats[ 9]-1] th9;
  ordered[ADASCats[10]-1] th10;
  ordered[ADASCats[11]-1] th11;
  ordered[ADASCats[12]-1] th12;
  row_vector[D] c0ADAS[N];            // Factor scores
  real<lower=0, upper=5> sigma_alpha;
  cholesky_factor_corr[D] L_corr_d;   // Cholesky correlation between factors
}
transformed parameters{
  // Auxiliary varaibles:
  //   velocity field parameters in the CSF+ADAS space
  matrix[K, K] v;
  matrix[K, K] wAge;
  matrix[K, K] wAPOE;
  row_vector[K] c0[N];
  v[1:kCSF, 1:kCSF] = vCSF;
  v[1:kCSF, (kCSF + 1):K] = zCSF;
  v[(kCSF + 1):K, :] = vADAS;
  wAge[1:kCSF, 1:kCSF] = wCSFAge;
  wAge[1:kCSF, (kCSF + 1):K] = zCSF;
  wAge[(kCSF + 1):K, :] = wADASAge;
  wAPOE[1:kCSF, 1:kCSF] = wCSFAPOE;
  wAPOE[1:kCSF, (kCSF + 1):K] = zCSF;
  wAPOE[(kCSF + 1):K, :] = wADASAPOE;

  for (k in 1:kCSF)
    c0[:, k] = to_array_1d(muRECSF[k] + c0CSF[k]*sRECSF[k]);
  c0[:, (1+kCSF):K] = c0ADAS;
```

```
}
model {
  matrix[n, K + m] mu;

  // Priors
  for (k in 1:kTau)
    sTau[k] ~ normal(0, 0.2);
  sAB ~ normal(0, 0.2);
  for (k2 in 1:kCSF) {
    bDX[k2] ~ normal(0, 100);
    for (k1 in 1:kCSF) {
      wCSFAge[k1, k2] ~ normal(0, 0.02);
      wCSFAPOE[k1, k2] ~ normal(0, 0.02);
      vCSF[k1, k2] ~ normal(0, 0.1);
    }
  }

  for (k in 1:K) {
    v0[k] ~ normal(0,0.1);
    for (d in 1:D) {
      wADASAge[d, k] ~ normal(0, 0.05);
      wADASAPOE[d, k] ~ normal(0, 0.05);
      vADAS[d, k] ~ normal(0, 0.1);
    }
  }

  for (k in 1:kCSF) {
    sRECSF[k] ~ std_normal();
    muRECSF[k] ~ std_normal();
  }
  for (i in 1:N)
    for (k in 1:kCSF)
      c0CSF[k, i] ~ std_normal();

  // priors: hierarchical (RE) for ADAS slopes
  sigma_alpha ~ std_normal();
  alpha ~ lognormal(0,sigma_alpha);
  // informative prior for ADAS latent variables
  L_corr_d ~ lkj_corr_cholesky(1);
  c0ADAS ~ multi_normal_cholesky(rep_vector(0, D), L_corr_d);

  // Trajectory
  for (i in 1:n) {
    matrix[K,1] c = scale_matrix_exp_multiply(t[i], (v + AGE[ID[i]]*wAge +
                                                  APOE4[ID[i]]*wAPOE),
                                     to_matrix( ( c0[ID[i]] - v0 ), K, 1));
    mu[i,1:K] = (to_row_vector(c) + v0);
  }
  mu[:,(K + 1):(K + m)] = X;

  // Likelihood
  // ABeta
  for (i in 1:nAB)
    AB[i] ~ normal(mu[idxAB[i],1], sAB);
  for (i in 1:NABCens) // Left censored ABeta
    target +=  normal_lcdf(ABmin | mu[idxABCens[i], 1], sAB);

  // Tau
```

```
  for (k in 1:kTau)
    for (i in 1:nTau)
      Tau[i,k] ~ normal(mu[idxTau[i],k+1], sTau[k]);

  //Diagnosis
  target += ordered_logistic_glm_lpmf(DX | mu[idxDX,:], bDX, cDX);

  //IRT ADAS-Cog
  Q1  ~ ordered_logistic(to_vector(mu[idxADAS, kCSF + it_d[1]] ) * alpha[1],  th1);
  Q2  ~ ordered_logistic(to_vector(mu[idxADAS, kCSF + it_d[2]] ) * alpha[2],  th2);
  Q3  ~ ordered_logistic(to_vector(mu[idxADAS, kCSF + it_d[3]] ) * alpha[3],  th3);
  Q4  ~ ordered_logistic(to_vector(mu[idxADAS, kCSF + it_d[4]] ) * alpha[4],  th4);
  Q5  ~ ordered_logistic(to_vector(mu[idxADAS, kCSF + it_d[5]] ) * alpha[5],  th5);
  Q6  ~ ordered_logistic(to_vector(mu[idxADAS, kCSF + it_d[6]] ) * alpha[6],  th6);
  Q7  ~ ordered_logistic(to_vector(mu[idxADAS, kCSF + it_d[7]] ) * alpha[7],  th7);
  Q8  ~ ordered_logistic(to_vector(mu[idxADAS, kCSF + it_d[8]] ) * alpha[8],  th8);
  Q9  ~ ordered_logistic(to_vector(mu[idxADAS, kCSF + it_d[9]] ) * alpha[9],  th9);
  Q10 ~ ordered_logistic(to_vector(mu[idxADAS, kCSF + it_d[10]]) * alpha[10], th10);
  Q11 ~ ordered_logistic(to_vector(mu[idxADAS, kCSF + it_d[11]]) * alpha[11], th11);
  Q12 ~ ordered_logistic(to_vector(mu[idxADAS, kCSF + it_d[12]]) * alpha[12], th12);
}
```

Figure S3: Stan code

References

[Gelman et al., 2013] Gelman, A., Carlin, J. B., Stern, H. S., Dunson, D. B., Vehtari, A., and Rubin, D. B. (2013). *Bayesian Data Analysis*. Chapman and Hall/CRC, third edition.

[Hodges, 2013] Hodges, J. S. (2013). *Richly Parameterized Linear Models: Additive, Time Series, and Spatial Models Using Random Effects*. Chapman and Hall/CRC, first edition.

[McCullagh, 1980] McCullagh, P. (1980). Regression models for ordinal data. *Journal of the Royal Statistical Society. Series B (Methodological)*, 42(2):109–142.

[Samejima, 1968] Samejima, F. (1968). Estimation of latent ability using a response pattern of graded scores. *ETS Research Bulletin Series*, 1968(1):i–169.

[Verma et al., 2015] Verma, N., Beretvas, S. N., Pascual, B., Masdeu, J. C., Markey, M. K., and Initiative, T. A. D. N. (2015). New scoring methodology improves the sensitivity of the Alzheimer's Disease Assessment Scale-Cognitive subscale (ADAS-Cog) in clinical trials. *Alzheimer's Research & Therapy*, 7(1):64.
